# DNA Barcoding Reveals High Levels of Divergence among Mitochondrial Lineages of *Brycon* (Characiformes, Bryconidae)

**DOI:** 10.3390/genes10090639

**Published:** 2019-08-23

**Authors:** Pábila S. S. Arruda, Daniela C. Ferreira, Claudio Oliveira, Paulo C. Venere

**Affiliations:** 1 Programa de Pós-Graduação em Ecologia e Conservação da Biodiversidade, Instituto de Biociências, Universidade Federal de Mato Grosso, Avenida Fernando Correia da Costa, 2367, Cuiabá, Mato Grosso 78060-900, Brazil; 2 Departamento de Biologia e Zoologia, Instituto de Biociências, Universidade Federal de Mato Grosso, Avenida Fernando Correia da Costa, 2367, Cuiabá, Mato Grosso 78060-900, Brazil; 3 Departamento de Morfologia, Instituto de Biociências, Universidade Estadual Paulista Júlio de Mesquita Filho—UNESP, Rubião Jr S-N, Botucatu, São Paulo 18618-970, Brazil

**Keywords:** Freshwater fish, MOTUs, biodiversity, Neotropical region, matrinchã

## Abstract

*Brycon* is an important group of Neotropical fish and the principal genus of the family Bryconidae, with 44 valid species that are found in some Central American rivers and practically all the major hydrographic basins of South America. These fish are medium to large in size, migratory, omnivorous, important seed dispersers for riparian forests, and bioindicators of environmental quality, given that they are found preferentially in rivers with clean, well oxygenated water. Many *Brycon* species are important fishery resources and some are farmed. Morphological and molecular studies have nevertheless indicated that the group is not monophyletic and has a number of unresolved taxonomic problems. Given this, the present study aimed to identify the Molecular Operational Taxonomic Units (MOTUs) of the genus using the mitochondrial cytochrome c oxidase I (COI) gene, with analyses of genetics distance (NJ), maximum likelihood (ML), and Bayesian Inference (BI), combined with two different species delimitation approaches (GMYC and ABGD). The results indicate that at least 31 MOTUs exist within the 18 species identified a priori based on their morphology. Many of these lineages require further investigation for a more definitive classification.

## 1. Introduction

The genus *Brycon* Müller & Troschel, 1844 is one of the most diverse groups of Neotropical fish of the order Characiformes, with 44 valid species [1]. The species of this genus are found to the west of the Andes, in rivers in Peru, Colombia, and Ecuador, and also east of this cordillera, in the basins of the Orinoco, Amazon, Tocantins-Araguaia, Plate, and São Francisco rivers, as well as most of the river systems of the Guianas, and the coastal rivers of eastern Brazil, between the mouth of the São Francisco River and the Paraíba do Sul basin [1,2].

*Brycon* are medium to large sized fish, with a total length of between 16 cm in *B. pesu* and 70 cm in *B. orbignyanus*. In Brazil, these fish have a number of common names, including matrinchã, jatuarana, piabanha, pirapitinga, piracanjuba, and piraputanga, while in English, they are sometimes referred to as South American trout [3,4]. These fish are migratory and are bioindicators of environmental quality, given that they prefer rivers with clean and highly oxygenated water [2,5]. *Brycon* are also important seed dispersers, but have an omnivorous diet, which includes invertebrates and small vertebrates, in addition to fruit and seeds [2,3,5,6,7].

The taxonomy and phylogeny of *Brycon* are still poorly defined, and up to now, it has not been possible to identify unique, derived characteristics that define the genus [1]. Given this, the analysis of molecular markers has provided increasingly valuable insights into the evolutionary relationships within the genus. One fundamentally important approach here is the biological identification system based on DNA sequences known as the DNA barcode, as proposed by Hebert et al. [8], which focuses on a short sequence of the mitochondrial DNA, which has become a global system for the identification of animal species, in particular, when the identification of the taxon based on morphological criteria is impossible or unreliable. 

Many recent studies have applied this approach to species identification and have proved its rapidity and reliability for the identification of taxa through species-specific criteria [8,9]. The DNA barcoding approach has been shown to be particularly effective for the delimitation of fish species, including both marine [9,10] and freshwater groups [11,12,13,14,15,16,17,18]. In addition to complementing traditional taxonomic methods, the DNA barcode provides a new perspective on fish species diversity, given the extreme morphological diversity of many forms, which often hampers the reliable diagnosis of species, as in the case of *Brycon*.

A recent review of *Brycon* [1] included the descriptions of three new species from South America, east of the Andes, *B. howesi*, *B. dulcis* and *B. vonoi*, and also invalidated a number of species, including *B. cephalus*, which was synonimized with *B. amazonicus*, and *B. bicolor* and *B. brevicauda*, which were synonimized with *B. falcatus*. Despite proposing this synonimization, the author also found that many of the specimens identified as *B. falcatus* varied considerably in size and the pigmentation of the caudal fins, which may reflect phenotypic plasticity. The author also concluded that this species is represented by two distinct morphotypes in the upper Tapajós and Xingu basins, which are subtly distinct in body size and the spotting of the adipose fin.

Given the morphological complexity in the species of *Brycon*, the present study adopted a molecular approach to identify the different Molecular Operational Taxonomic Units (MOTUs) found in the hydrographic basins of the Neotropical region. This study was based on a number of different approaches for species delimitation.

## 2. Materials and Methods

### 2.1. Study Area and Sample Collection

All the tissue samples and voucher specimens analyzed in the present study were collected during previous surveys of a number of different hydrographic basins in South and Central America (Table S1) and were acquired with the collaboration of researchers from a number of different Brazilian institutions. Samples from a total of 350 individuals were collected, representing 18 *Brycon* species defined based on morphological characteristics. Some of the voucher specimens are available in the fish collection of the Federal University of Mato Grosso (CPUFMT) and the Laboratory of Fish Biology and Genetics (LBP) of the Paulista State University in Botucatu, São Paulo, Brazil. In addition, two other Bryconidae species were used in the analyses, one belonging to *Henochilus* and one to *Chilobrycon*. Two *Salminus* species (BOLD systems accession numbers BSB477-10 and BSB150-10) were included as outgroup.

### 2.2. Extraction of the DNA, Amplification, Purification, and Sequencing

The total genomic DNA was extracted using the saline extraction protocol [19]. Partial sequences of the cytochrome c oxidase I gene (COI) were amplified with the Fish F1 (5’-TCAACCAACCACAAAGACATTGGCAC-3’) and Fish R1 (5’-TAGACTTCTGGGTG GCCAAAGAATCA-3’) primers [9].

The Polymerase Chain Reaction (PCR) was prepared in a final volume of 25 µL, containing 1.5 μL of dNTP (1.25 mM), 2.5 μL of 10× buffer, 0.5 μL of the COI Fish-F1 primer (10 mM), 0.5 μL of the COI Fish-R1 primer (10 mM), 1.0 μL of MgCl2 (50 mM), 0.2 μL of Taq DNA Polymerase (Ludwig, 5 U/μL), 1 μL of DNA, and ultrapure water to complete the final volume. The reactions were processed in a thermocycler (Applied Byosystems, Veriti), with initial denaturation at 94 °C for 5 min, followed by 35 cycles of denaturation at 94 °C for 45 s, annealing at 50 °C for 60 s, and extension at 72 °C for 60 s, with a final extension at 72 °C for 5 min.

The amplified PCR products were visualized in 1% agarose gel and purified by the enzymatic method using Illustra ExoProStar enzymes (GE Healthcare Life Sciences, Buckinghamshire, UK). The fragments were sequenced in an ABI 3500 automatic sequencer (Applied Biosystems^®^, Austin, TX, USA), supplied by Myleus Biotechnology (www.myleus.com), Belo Horizonte, MG, Brazil.

### 2.3. Data Analysis

The Geneious R9 program [20] was used to define the consensus sequences and verify the presence of stop codons. The sequences were aligned using the MUSCLE algorithm [21]. The Kimura-2-Parameter (K2P) model of nucleotide substitution was used to determine the intra- and interspecific genetic distances, based on analyses run in MEGA v7.0 [22]. The patterns of divergence indicated by the genetic distances were plotted in a dendrogram based on the Neighbor-Joining (NJ) algorithm, with 1000 bootstrap replicates [23].

The Optimum Threshold (OT) was calculated from the data matrix and used for the delimitation of species. The LocalMinima function, which is based on the barcode gap concept, was applied here, using the SPIDER (SPecies IDentity and Evolution) package [24] of the R software (http://www.R-project.org). 

The Generalized Mixed Yule Coalescence (GMYC) phylogenetic model was used to delimit the species groups and, subsequently, the identification of the MOTUs [25]. An ultrametric tree was generated in BEAST v.2.4.6 [26], with the (TIM3+I+G) substitution model calculated in JModelTest 2.1.7 [27], using a relaxed molecular clock with a lognormal distribution and the birth–death model. Three independent runs of 50 million generations were conducted, and the runs were subsequently combined in LogCombiner v.2.4.6 [28]. The first 25% of the sampling was discarded. The data mix and the Effective Sample Size (ESS) were verified in Tracer v.1.6. [29]. The delimitation of species by GMYC was run on the (species.h-its.org/gmyc) web server using the single limit method. 

The Automatic Barcode Gap Discovery (ABGD) method was used to analyze genetic distances in the (http://wwwabi.snv.jussieu.fr/public/abgd/) web interface, using the Kimura 2-Parameter (K2P) model of nucleotide substitution, at the default setting [30]. Bayesian Inference (BI) was run in MrBayes 3.1.2 [31] with four chains, 50 million generations, trees sampled every 1000 generations and 25% burn-in. Maximum likelihood analysis (ML) was performed in the Garli 2.0 program [32]. Clade support was estimated based on 1000 bootstrap pseudoreplicates. The trees obtained in the Garli program were summarized by majority consensus in the SumTrees program. The ML and BI trees were visualized and edited in Figtree, version 1.4.3 [33].

Further analysis of populations genetics were run in three cases, i.e., *B. falcatus*, *B. pesu* and *B. amazonicus*, to identify and interpret the levels of genetic variation found among the MOTUs identified in these species. The number of haplotypes, and the indices of haplotype (Hd) and nucleotide (π) diversity were obtained in the DnaSP v5 (DNA Sequence Polymorphism) program [34]. The relationships among the different haplotypes were evaluated using a haplotype network constructed using the Median—Joining [35] method, run in PopART 1.7 [36]. The genetic differentiation of the MOTUs was determined by the results of an Analysis of Molecular Variance (AMOVA) run in Arlequin 3.5 [37].

## 3. Results

### 3.1. Species Delimitation

**Figure 1:**
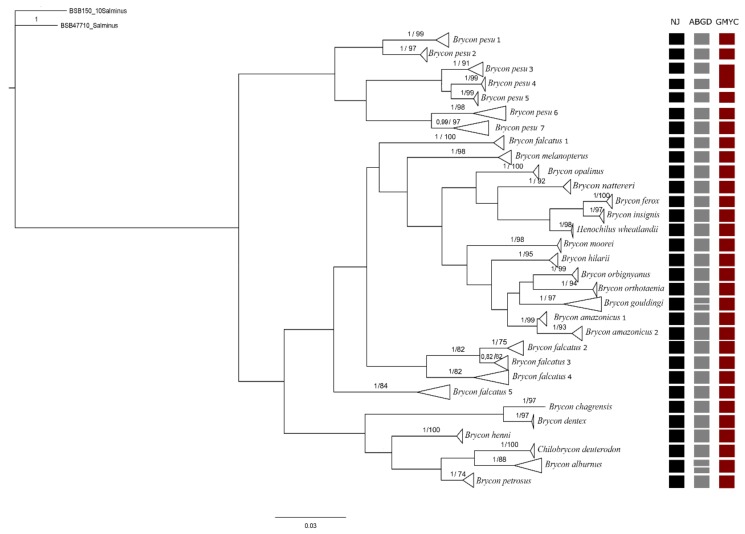
Bayesian Inference topology based on the cytochrome c oxidase I (COI) gene. The bars at the side of the tree represents the results of the analyses of species delimitation. The numbers next to the nodes represent the posterior probability/bootstrap values of the ML analyses.

The final data matrix used for analysis was composed of 350 sequences of 597 bases. No saturation in nucleotide substitution by either transition (R² = 0.9259) or transversion (R² = 0.7874) was identified. The Bayesian Inference (BI) analysis identified a total of 31 MOTUs in the 20 study species (18 *Brycon* species, *Henochilus wheatlandii,* and *Chilobrycon deuterodon*), defined a priori, based on morphological criteria, with an OT of genetic divergence of 0.0182 (or 1.82%), and high statistical support for both the ML and the BI analyses. All other analyses produced similar results. NJ identified 31 MOTUs (Figure S1). ML identified 30 MOTUs, *B*. *falcatus* 2 and 3 MOTUs were not differentiated statistically (Figure S2). The GMYC analyses only recovered 30 MOTUs (Figure S3), however, this is due to the fact that the *B. pesu* MOTUs were not differentiated statistically (referred to here as samples 3 and 4). By contrast, the ABGD analysis recovered 33 MOTUs, including two each in *B. gouldingi* and *B. alburnus* (Figure 1). 

**Figure 2:**
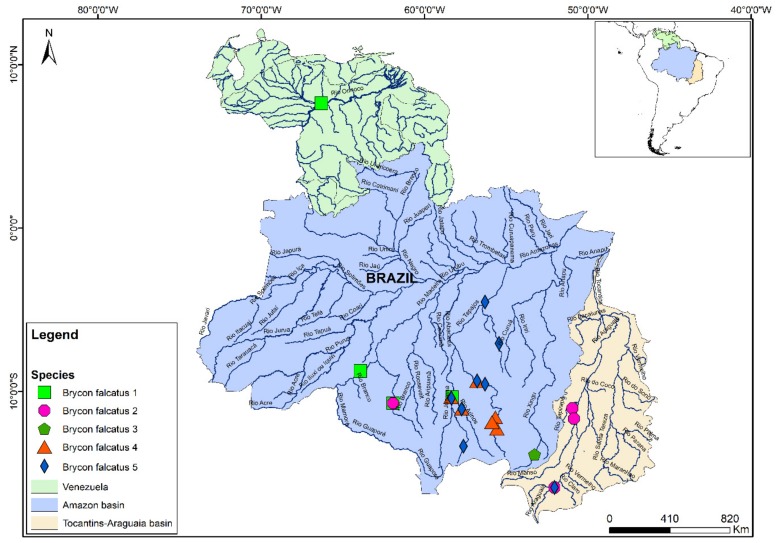
Distribution of the Molecular Operational Taxonomic Units (MOTUs) of *Brycon falcatus* identified in the molecular analyses.

According to the combined analyses, the species identified initially as *B. falcatus* is composed of five distinct lineages: *B. falcatus* lineage 1, from the Orinoco basin, and the Madeira and Juruena rivers; *B. falcatus* lineage 2, from the Tocantins-Araguaia basin; *B. falcatus* lineage 3, from the Culuene River, in the Xingu basin; *B. falcatus* lineage 4, from the Teles Pires, Juruena, Arinos, and Rio Verde rivers, which are all tributaries of the Tapajós, and *B. falcatus* lineage 5, from the Juruena, Arinos, and Teles Pires rivers (Tapajós basin), and the Araguaia River (Figure 2). The greatest interspecific genetic distance was recorded between *B. falcatus* lineages 1 and 2 (0.143 ± 0.019), that is, 14.3%, whereas the smallest distance was 0.028 ± 0.008 (2.8%), between *B. falcatus* lineages 2 and 3 (Table S2). 

**Figure 3:**
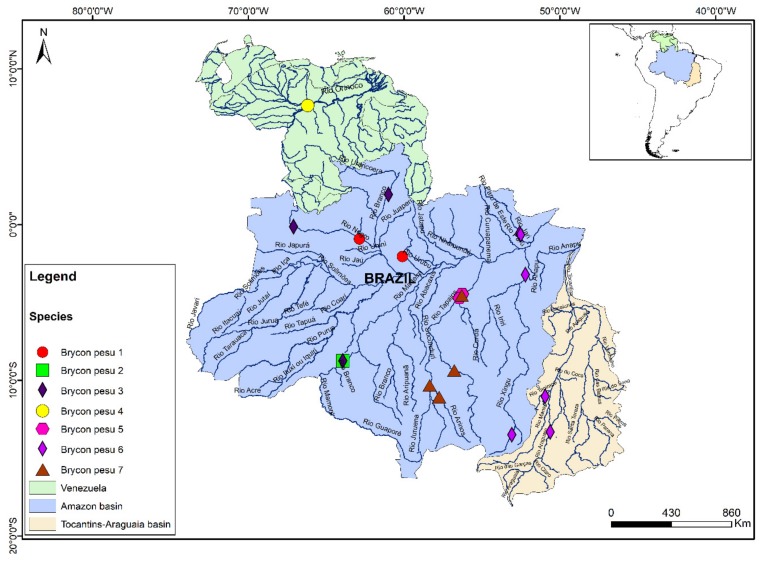
Distribution of the MOTUs of *Brycon pesu* identified in the molecular analyses.

In *B. pesu*, the NJ and ABGD analyses indicated the existence of seven distinct lineages: *B. pesu* lineage 1, from the Tarumã and Negro rivers, in the Negro basin; *B. pesu* lineage 2, from the Madeira River; *B. pesu* lineage 3, from the Madeira, Negro, and Branco rivers; *B. pesu* lineage 4, from the Orinoco River; *B. pesu* lineage 5, from the Tapajós, Tracuá, and Igarapé (Tapajós basin); *B. pesu* lineage 6, from the Araguaia and Xavantinho rivers (Tocantins-Araguaia basin), the Culuene and Xingu rivers (Xingu basin), and the Iratapuru and Jari rivers, in the Amazon basin, and *B. pesu* lineage 7, from the Juruena, Arinos, and Teles Pires rivers in the Tapajós basin (Figure 3). The greatest interspecific distance (12.4%) was recorded between *B. pesu* lineages 2 and 3 (0.124 ± 0.017), while the smallest distance (2.8%) was found between *B. pesu* lineages 4 and 5, that is, 0.028 ± 0.007 (Table S2). In the GMYC analysis, however, *B. pesu* lineages 3 and 4 were considered to form a single MOTU. In this case, the threshold time was −0.0087, which indicates that the lineages have yet to undergo a speciation. Negative threshold values indicate diversification events. The probability of the null model was 1044.005, and the ML of the GMYC model was 1061.177. *B. amazonicus* was also represented by two lineages, separated by a genetic distance of 0.023 ± 0.007 (2.3%) between the samples from the Amazon and Orinoco basins (Figure 1).

The ABGD analysis identified seven partitions. Partition 1 was made up of 93 MOTUs, while partition 7 had only a single MOTU. Partition 6 (Prior maximal distance *p* = 0.012) best represented the data, and identified 33 distinct lineages, with additional lineages being recognized in *B. gouldingi* and *B. petrosus*, resulting in a larger number of MOTUs than that recorded in the other analyses. 

### 3.2. Diversity Genetic of the B. falcatus, B. pesu, and B. amazonicus Lineages

**Figure 4:**
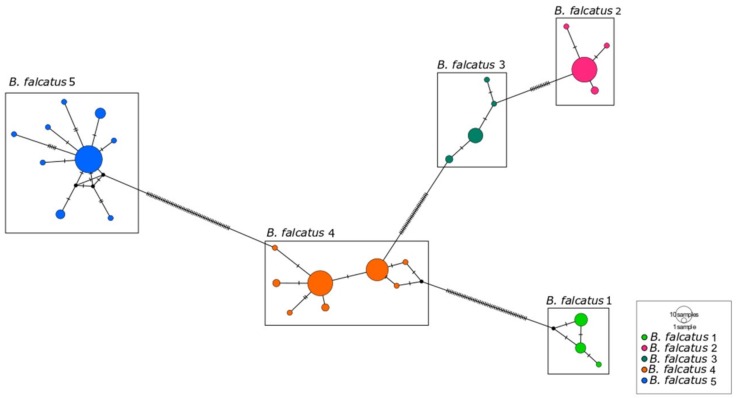
Haplotype network of the five mitochondrial lineages (MOTUs) identified in *Brycon falcatus.* Each circle represents a single haplotype and its size is proportional to its frequency.

The haplotype network of *B. falcatus*, based on 139 sequences of 540 base pairs (bps) included 28 haplotypes (Figure 4), with haplotype diversity (Hd) of 0.888 ± 0.012 and nucleotide diversity (π) of 0.07410 ± 0.002. The results of the AMOVA indicates that greater variation exists between the lineages/groups (MOTUs) of *B. falcatus* (96.72%) than within these groups (1.75%) or within populations (1.53%) (Table 1).

**Figure 5:**
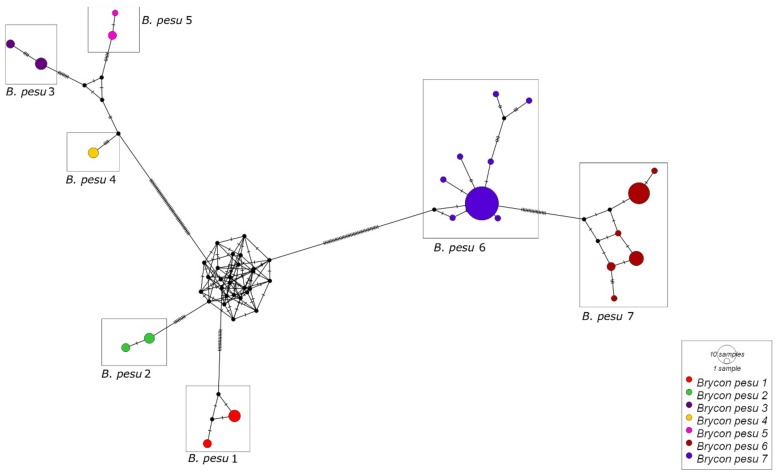
Haplotype network of the seven mitochondrial lineages (MOTUs) identified in *Brycon pesu.* Each circle represents a single haplotype and its size is proportional to its frequency.

The haplotype network of *B. pesu* was based on 86 sequences of 589 bps, which generated 23 haplotypes (Figure 5), with a haplotype diversity (Hd) of 0.883 ± 0.034 and nucleotide diversity (π) of 0.05812 ± 0.005. As in *B. falcatus* most of the variation (AMOVA: 91.96%) was found between lineages (Table 2).

**Figure 6:**
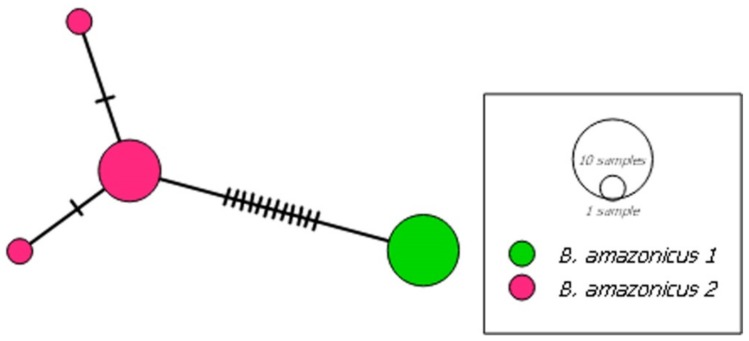
Haplotype network of the two mitochondrial lineages (MOTUs) identified in *Brycon amazonicus*. Each circle represents a single haplotype and its size is proportional to its frequency.

The haplotype network of *B. amazonicus* was based on 16 sequences of 589 bps, which were arranged in four haplotypes (Figure 6), with haplotype diversity (Hd) of 0.642 ± 0.081 and nucleotide diversity (π) of 0.01129 ± 0.00096. As in the other two *Brycon* species (Table 1 and Table 2), most of the variation found in the AMOVA (97.52%) was between the two lineages (Table 3).

## 4. Discussion

The species delimitation analyses presented here recognized a larger number of genetic lineages than the 20 species identified a priori, based on morphological criteria. In particular, *B*. *falcatus*, *B*. *pesu*, and *B*. *amazonicus* are represented by two or more MOTUs, separated by a high degree of genetic diversity. A number of previous studies of genetic species delimitation in Neotropical fish have produced similar findings, in which the nominal species identified on the basis of morphological characteristics were recovered as distinct molecular taxonomic units [13,16,38,39,40,41,42,43,44,45]. 

The discrimination of species based on partial COI sequences requires that the intraspecific genetic variation is lower than that found between species. A 2% threshold was proposed by [46] for the delimitation of fish species. The threshold identified in the present study (1.82%) is consistent with this assumption, given that the same number of MOTUs was obtained, whether a 2% or a 1.82% threshold was considered (Table S2).

The GMYC, ABGD, BI, and ML approaches have been applied increasingly for the delimitation of species, although some studies [44,45,47,48] have recovered a larger number of lineages using the GMYC approach in comparison with the other methods. The results of the present study contrasted with this pattern, however, given that the GMYC analysis did not separate *B. pesu* lineages 3 and 4 into distinct MOTUs, even though they were separated by genetic distances of over 1.82% (3.2%). While the genetic distance between these lineages was greater than the OT, these MOTUs are found in distinct basins, with *B. pesu* lineage 4 in the Orinoco basin, and *B. pesu* lineage 3 from the Madeira, Negro, and Branco rivers. In addition, the ABGD analysis revealed a larger number of lineages due to the division of *B. gouldingi* and *B. alburnus* into two lineages each, despite the fact that the genetic distances are relatively small (0.1% and 1%, respectively) in comparison with the OT.

In all the analyses conducted in the present study, the clade of the *Brycon* lineages from the east of the Andes included *Chilobrycon*, which is consistent with Abe et al. [49] phylogenetic analysis of the Bryconidae, and reinforces the hypothesis that the genus *Brycon* is in fact a paraphyletic group. Travenzoli et al. [50] obtained similar results in an integrated study of approximately 15 bryconine species, in which they identified *Henochilus* as the sister group of *B. ferox*, further reinforcing the paraphyletism of the genus. The most basal species in the analysis of Abe et al. [49] were those from the west of the Andes, that is, *B. henni*, *B. petrosus* and *B. chagrensis*. In the present study, *B. chagrensis*, *B. dentex*, *B. henni*, *B. alburnus*, *B. petrosus*, and *Chilobrycon deuterodon*, which all occur to the east of the Andes, were also grouped together.

In the specific case of *B. falcatus*, in which five MOTUs were identified in the present study, it is important to note that Abe et al. had already identified at least two distinct clades, which they referred to as *B. falcatus*, from the Orinoco River, and *B.* cf. *falcatus*, from the Xingu [49]. These authors also examined specimens identified as *B. pesu* and concluded that this taxon did in fact constitute a species complex [1,49]. The results of the present study further reinforce these previous findings and point to the existence of cryptic species (or misidentified taxa) within the populations assigned to *B. falcatus* and *B. pesu*. 

Based on its morphology, *B. falcatus* had previously been recognized as an amply distributed species, present in many of the freshwater basins of the Neotropical region. More recently, however, a number of studies have shown that this group is characterized by considerable morphological and meristic variation, which hampers the determination of the possible taxonomic units [1], and the identification of valid species, even by specialists. It is also important to note here that, while many MOTUs are found in distinct drainage basins, some are found within the same hydrographic system, that is, samples collected from the same basins that represented distinct, sympatric taxonomic units, as in the case of *B. falcatus* lineages 1, 4, and 5 from the Tapajós River. A similar situation was confirmed in the *B. pesu* species complex, where *B. pesu* lineages 2 and 3 are sympatric in the Madeira River, and *B. pesu* lineages 5 and 7 are sympatric in the Tapajós basin.

The level of genetic differentiation found in the *B. falcatus* lineages by the AMOVA also confirmed the differences found among the MOTUs, indicating that they are structured, as observed in the haplotype network (Figure 4). The geographic differentiation of populations is even more apparent, then, when the differences among haplotypes are taken into account. It was also possible to confirm that the molecular distances between pairs of haplotypes from different basins are much greater than those recorded within a given region [51]

*B. pesu*, as defined currently, has an ample geographic distribution, with records from the Amazon, Tocantins-Araguaia, and Orinoco basins, and the river systems of Guyana, Suriname, and French Guiana. As in the case of *B. falcatus*, however, this ample distribution may simply reflect the inadequate classification of this group. In fact, several authors have previously proposed that *B. pesu* represents a species complex, composed of a number of different taxonomic units that have yet to be described as valid species [1]. This complex appears to have passed through a rapid process of diversification over the past 6.7 ± 2.0 million years, a period that coincides with the formation of the Amazon and Orinoco basins [49]. The results of the present study are consistent with this viewpoint and confirm this hypothesis, given the identification of at least seven distinct taxonomic units, with both allopatric and sympatric MOTUs. The ample genetic divergence among the MOTUs was confirmed by the AMOVA, and in particular, by the *B. pesu* haplotype network (Figure 5).

In addition to *B. falcatus* and *B. pesu, B. amazonicus* is also represented by two distinct MOTUs, one from the Amazon basin (*B. amazonicus* lineage 1) and the other (*B. amazonicus* lineage 2) from the Orinoco River, which were separated by a mean genetic distance of 2.3%, which is within the OT and indicates that the taxon may in fact include two valid species, *B*. *amazonicus* and a second, as yet unidentified taxon. 

Although the data reveal candidate species, it is important to emphasize that analysis based on only one gene is not conclusive, but represents an important step in studies on species delimitation. In this way, the findings of the present study reinforce the need for a more detailed morphological analysis, integrated with molecular data from more informative genes, for the identification of the potential new species (MOTUs) identified here. The data obtained in the present study also represent an important source of reference for the understanding of the mechanisms involved in the genetic diversification of this important, and highly complex group of Neotropical fish.

## Figures and Tables

**Table 1:** Analysis of Molecular Variance (AMOVA) of the samples of *Brycon falcatus*.

Source of the Variation	d.f.	Sum of Squares	Components of the Variance	% Variation
**Among groups**	4	2626.838	25.06425 Va	96.72
**Among populations within groups**	3	7.016	0.45465 Vb	1.75
**Within populations**	131	51.779	0.39526 Vc	1.53

**Table 2:** Analysis of Molecular Variance (AMOVA) of the samples of *Brycon pesu*.

Source of the Variation	d.f.	Sum of Squares	Components of the Variance	% Variation
**Among groups**	6	1271.181	20.05707 Va	91.96
**Among populations within groups**	3	20.335	1.21388 Vb	5.57
**Within populations**	76	41.055	0.54019 Vc	2.48

**Table 3:** Analysis of Molecular Variance (AMOVA) of the samples of *Brycon amazonicus.*

Source of the Variation	d.f.	Sum of Squares	Components of the Variance	% Variation
**Among groups**	1	48.125	5.97295 Va	97.52
**Among populations within groups**	2	0.417	0.04094 Vb	0.67
**Within populations**	12	1.333	0.11111 Vc	1.81

## Supplementary Materials

The following are available online at https://www.mdpi.com/2073-4425/10/9/639/s1, Table S1: General table of data about the sampled species, Table S2: Intra and interspecific distance among detected MOTUS, Figure S1: Neighbor joining tree based on the cytochrome c oxidase I gene for *Brycon*, Figure S2: Maximum likelihood tree based on the cytochrome c oxidase I gene for *Brycon* species, Figure S3: GMYC tree based on the cytochrome c oxidase I gene for *Brycon* species.
