# Supplementary material for: DNA Barcoding Reveals High Levels of Divergence among Mitochondrial Lineages of Brycon (Characiformes, Bryconidae)
Source: Genes (Basel). 2019 Aug 23;10(9):639. doi: 10.3390/genes10090639 (PMC6769914; doi:10.3390/genes10090639)
Supplement: Supplementary file 1 [file genes-10-00639-s001.zip › Tab S2 genetic distance .pdf]

	1	2	3	4	5	6	7	8	9	10	11	12	13	14	15	16	17	18	19	20	21	22	23	24	25	26	27	28	29	30	31	32
**1.** *B. pesu* 1	**0,002**	0,011	0,017	0,017	0,016	0,018	0,017	0,024	0,024	0,023	0,022	0,022	0,022	0,022	0,023	0,022	0,023	0,024	0,024	0,023	0,024	0,025	0,024	0,023	0,024	0,024	0,024	0,021	0,020	0,021	0,020	0,027
**2.** *B. pesu* 2	0,057	**0,001**	0,017	0,017	0,016	0,019	0,017	0,022	0,024	0,024	0,022	0,021	0,021	0,020	0,021	0,020	0,020	0,020	0,024	0,021	0,022	0,022	0,023	0,021	0,022	0,025	0,026	0,022	0,022	0,023	0,020	0,026
**3.** *B. pesu* 3	0,127	0,124	**0,004**	0,008	0,008	0,016	0,015	0,023	0,026	0,026	0,024	0,024	0,022	0,025	0,023	0,023	0,023	0,024	0,025	0,022	0,024	0,023	0,023	0,023	0,024	0,024	0,024	0,022	0,024	0,025	0,023	0,023
**4.** *B. pesu* 4	0,126	0,128	0,032	**0,000**	0,007	0,017	0,016	0,022	0,026	0,026	0,024	0,023	0,022	0,024	0,023	0,023	0,024	0,024	0,025	0,022	0,024	0,022	0,023	0,023	0,024	0,024	0,024	0,022	0,023	0,025	0,022	0,024
**5.** *B. pesu* 5	0,125	0,122	0,033	0,028	**0,001**	0,016	0,016	0,022	0,025	0,025	0,024	0,023	0,023	0,024	0,022	0,023	0,024	0,024	0,024	0,022	0,023	0,022	0,023	0,021	0,022	0,025	0,024	0,022	0,023	0,025	0,022	0,024
**6.** *B. pesu* 6	0,127	0,135	0,113	0,118	0,117	**0,004**	0,009	0,021	0,024	0,024	0,022	0,023	0,022	0,024	0,026	0,023	0,023	0,024	0,024	0,023	0,023	0,023	0,022	0,022	0,022	0,026	0,025	0,023	0,026	0,026	0,024	0,027
**7.** *B. pesu* 7	0,122	0,118	0,111	0,116	0,114	0,042	**0,001**	0,022	0,024	0,024	0,022	0,022	0,021	0,023	0,024	0,022	0,021	0,022	0,023	0,021	0,022	0,022	0,022	0,021	0,023	0,026	0,025	0,023	0,024	0,025	0,022	0,026
**8.** *B. falcatus* 1	0,210	0,190	0,195	0,200	0,200	0,183	0,192	**0,002**	0,019	0,019	0,017	0,020	0,018	0,019	0,019	0,019	0,019	0,019	0,019	0,019	0,018	0,019	0,018	0,017	0,018	0,026	0,026	0,020	0,020	0,021	0,019	0,026
**9.** *B. falcatus* 2	0,208	0,218	0,236	0,237	0,231	0,210	0,214	0,143	**0,001**	0,008	0,013	0,019	0,016	0,017	0,018	0,018	0,020	0,020	0,019	0,019	0,018	0,019	0,017	0,018	0,019	0,023	0,022	0,022	0,023	0,022	0,021	0,027
**10.** *B. falcatus* 3	0,207	0,220	0,232	0,227	0,220	0,212	0,216	0,142	0,028	**0,002**	0,012	0,019	0,017	0,019	0,020	0,020	0,020	0,020	0,019	0,020	0,020	0,020	0,018	0,020	0,020	0,024	0,022	0,021	0,023	0,023	0,021	0,026
**11.** *B. falcatus* 4	0,191	0,193	0,206	0,212	0,206	0,188	0,189	0,117	0,076	0,064	**0,002**	0,017	0,016	0,019	0,019	0,020	0,019	0,020	0,018	0,018	0,020	0,019	0,019	0,019	0,019	0,024	0,024	0,019	0,022	0,022	0,020	0,028
**12.** *B. falcatus* 5	0,187	0,185	0,208	0,194	0,198	0,198	0,187	0,146	0,133	0,136	0,122	**0,002**	0,019	0,017	0,018	0,018	0,018	0,018	0,019	0,018	0,018	0,018	0,018	0,017	0,018	0,024	0,024	0,020	0,020	0,021	0,017	0,025
**13.** *B. melanopterus*	0,186	0,174	0,185	0,181	0,187	0,176	0,166	0,136	0,116	0,120	0,123	0,141	**0,001**	0,018	0,018	0,016	0,017	0,017	0,015	0,017	0,017	0,017	0,017	0,017	0,018	0,023	0,025	0,021	0,022	0,023	0,021	0,026
**14.** *B. opalinus*	0,181	0,165	0,221	0,202	0,216	0,198	0,193	0,147	0,124	0,143	0,141	0,127	0,121	**0,000**	0,014	0,015	0,016	0,016	0,017	0,015	0,014	0,015	0,015	0,014	0,014	0,021	0,020	0,022	0,021	0,023	0,020	0,025
**15.** *B. nattereri*	0,201	0,186	0,205	0,191	0,193	0,233	0,213	0,137	0,140	0,151	0,147	0,139	0,132	0,081	**0,001**	0,013	0,015	0,015	0,017	0,016	0,015	0,017	0,016	0,015	0,015	0,022	0,022	0,020	0,021	0,022	0,020	0,024
**16.** *H. wheatlandii*	0,180	0,165	0,194	0,186	0,192	0,195	0,189	0,152	0,132	0,152	0,149	0,130	0,113	0,090	0,079	**0,000**	0,010	0,010	0,017	0,016	0,016	0,017	0,015	0,015	0,015	0,021	0,022	0,021	0,021	0,022	0,019	0,025
**17.** *B. insignis*	0,194	0,165	0,203	0,207	0,216	0,205	0,187	0,150	0,161	0,165	0,154	0,138	0,134	0,112	0,103	0,051	**0,004**	0,007	0,017	0,017	0,016	0,017	0,016	0,015	0,016	0,023	0,023	0,023	0,022	0,023	0,020	0,024
**18.** *B. ferox*	0,197	0,168	0,212	0,210	0,216	0,214	0,190	0,147	0,158	0,162	0,155	0,140	0,126	0,110	0,098	0,051	0,025	**0,000**	0,018	0,017	0,017	0,018	0,016	0,016	0,017	0,022	0,022	0,023	0,022	0,022	0,019	0,024
**19.** *B. moorei*	0,206	0,208	0,211	0,221	0,218	0,207	0,204	0,138	0,140	0,142	0,139	0,149	0,107	0,117	0,124	0,118	0,125	0,130	**0,000**	0,015	0,016	0,016	0,015	0,015	0,015	0,023	0,022	0,021	0,020	0,020	0,019	0,025
**20.** *B. hilarii*	0,200	0,181	0,192	0,188	0,192	0,197	0,183	0,145	0,150	0,160	0,147	0,143	0,128	0,107	0,112	0,110	0,127	0,127	0,108	**0,000**	0,012	0,012	0,013	0,012	0,014	0,023	0,022	0,019	0,020	0,021	0,018	0,025
**21.** *B. orbignyanus*	0,215	0,190	0,211	0,209	0,206	0,198	0,186	0,133	0,134	0,154	0,149	0,141	0,123	0,092	0,109	0,107	0,122	0,128	0,107	0,076	**0,000**	0,009	0,010	0,010	0,012	0,023	0,022	0,022	0,022	0,024	0,021	0,024
**22.** *B. gouldingi*	0,219	0,190	0,200	0,195	0,195	0,195	0,184	0,147	0,141	0,155	0,145	0,140	0,117	0,098	0,126	0,123	0,130	0,133	0,111	0,075	0,047	**0,001**	0,011	0,010	0,013	0,023	0,023	0,023	0,021	0,023	0,020	0,024
**23.** *B. orthotaenia*	0,215	0,198	0,205	0,202	0,200	0,197	0,191	0,146	0,129	0,138	0,141	0,144	0,132	0,104	0,117	0,105	0,114	0,119	0,105	0,084	0,053	0,063	**0,000**	0,011	0,012	0,022	0,022	0,022	0,024	0,024	0,021	0,024
**24.** *B. amazonicus* 1	0,200	0,173	0,201	0,194	0,183	0,189	0,181	0,131	0,134	0,144	0,134	0,128	0,122	0,083	0,102	0,098	0,107	0,115	0,103	0,069	0,053	0,054	0,060	**0,000**	0,007	0,024	0,023	0,022	0,021	0,022	0,019	0,024
**25.** *B. amazonicus* 2	0,208	0,187	0,210	0,203	0,192	0,195	0,201	0,139	0,135	0,144	0,141	0,141	0,123	0,088	0,105	0,103	0,117	0,130	0,096	0,091	0,072	0,080	0,074	0,023	**0,001**	0,023	0,023	0,022	0,021	0,022	0,019	0,024
**26.** *B. chagrensis*	0,222	0,231	0,215	0,217	0,223	0,242	0,253	0,234	0,200	0,206	0,207	0,212	0,208	0,185	0,197	0,188	0,207	0,197	0,199	0,205	0,204	0,208	0,182	0,213	0,204	**n/c**	0,010	0,019	0,022	0,021	0,020	0,022
**27.** *B. dentex*	0,222	0,231	0,218	0,222	0,226	0,227	0,238	0,222	0,186	0,180	0,202	0,215	0,220	0,169	0,186	0,182	0,213	0,202	0,179	0,196	0,195	0,205	0,180	0,199	0,196	0,045	**0,000**	0,019	0,023	0,022	0,020	0,022
**28.** *B. henni*	0,185	0,193	0,200	0,194	0,201	0,209	0,203	0,169	0,183	0,181	0,165	0,159	0,185	0,188	0,174	0,182	0,209	0,205	0,180	0,169	0,196	0,206	0,194	0,194	0,189	0,151	0,148	**0,000**	0,016	0,015	0,013	0,026
**29.** *C. deuterodon*	0,179	0,198	0,226	0,220	0,215	0,235	0,220	0,170	0,199	0,205	0,192	0,167	0,187	0,180	0,174	0,191	0,193	0,194	0,174	0,174	0,202	0,192	0,209	0,183	0,184	0,178	0,186	0,112	**0,000**	0,012	0,012	0,024
**30.** *B. alburnus*	0,194	0,207	0,242	0,236	0,237	0,248	0,236	0,170	0,194	0,203	0,188	0,171	0,199	0,195	0,190	0,197	0,205	0,194	0,169	0,182	0,208	0,205	0,205	0,188	0,183	0,179	0,180	0,106	0,068	**0,010**	0,011	0,023
**31.** *B. petrosus*	0,172	0,167	0,212	0,201	0,201	0,212	0,195	0,163	0,172	0,172	0,159	0,131	0,174	0,159	0,164	0,167	0,178	0,164	0,168	0,156	0,180	0,176	0,176	0,162	0,166	0,157	0,154	0,081	0,070	0,060	**0,001**	0,023
**32.** Grupo externo	0,260	0,254	0,222	0,225	0,225	0,273	0,263	0,252	0,260	0,250	0,278	0,255	0,246	0,249	0,238	0,240	0,234	0,239	0,237	0,252	0,246	0,247	0,232	0,243	0,230	0,216	0,222	0,258	0,247	0,222	0,233	**0,063**
