# Supplementary material for: DNA Barcoding Reveals High Levels of Divergence among Mitochondrial Lineages of Brycon (Characiformes, Bryconidae)
Source: Genes (Basel). 2019 Aug 23;10(9):639. doi: 10.3390/genes10090639 (PMC6769914; doi:10.3390/genes10090639)
Supplement: Supplementary file 1 [file genes-10-00639-s001.zip › Table S1.pdf]

Table S1: Bryconinae species analyzed, locality information, deposit and BOLD accession number.

Taxon	Voucher	Collection Code	City/State	Locality	Country	Accession Bold
B. alburnus	76044	19341	Machala/El Oro	Rio Dos Bocas	Equador	BRY220-19
B. alburnus	76045	19341	Machala/El Oro	Rio Dos Bocas	Equador	BRY221-19
B. alburnus	76096	19363	Machala/El Oro	Rio Dos Bocas	Equador	BRY217-19
B. alburnus	76099	19363	Machala/El Oro	Rio Dos Bocas	Equador	BRY218-19
B. alburnus	76100	19363	Machala/El Oro	Rio Dos Bocas	Equador	BRY219-19
B. alburnus	76109	19334	Babahoyo/Los Ríos	Rio Babahoyo	Equador	BRY222-19
B. alburnus	76110	19334	Babahoyo/Los Ríos	Rio Babahoyo	Equador	BRY223-19
B. amazonicus	8566	CPUFMT4497	Barra do Garças/MT	Rio Araguaia	Brazil	BRY093-16
B. amazonicus	8832	834	Manaus/AM	Rio Negro	Brazil	BRY224-19
B. amazonicus	8833	834	Manaus/AM	Rio Negro	Brazil	BRY225-19
B. amazonicus	15567	2187	Caicara del Orinoco /Bolivar	Rio Orinoco	Venezuela	BRY366-19
B. amazonicus	19122	3026	Caicara del Orinoco/Bolivar	Rio Orinoco	Venezuela	BRY229-19
B. amazonicus	19123	3026	Caicara del Orinoco/Bolivar	Rio Orinoco	Venezuela	BRY227-19
B. amazonicus	19186	3026	Caicara del Orinoco/Bolivar	Rio Orinoco	Venezuela	BRY228-19
B. amazonicus	43145	10224	Cabruta/Guárico	Rio Apure	Venezuela	BRY234-19
B. amazonicus	43146	10224	Cabruta/Guárico	Rio Apure	Venezuela	BRY235-19
B. amazonicus	43147	10224	Cabruta/Guárico	Rio Apure	Venezuela	BRY236-19
B. amazonicus	43148	10224	Cabruta/Guárico	Rio Apure	Venezuela	BRY237-19
B. amazonicus	46517	10143	Porto Velho/RO	Rio Abunã	Brazil	BRY230-19
B. amazonicus	46518	10143	Porto Velho/RO	Rio Abunã	Brazil	BRY231-19
B. amazonicus	46519	10144	Porto Velho/RO	Rio Abunã	Brazil	BRY232-19
B. amazonicus	46520	10143	Porto Velho/RO	Rio Abunã	Brazil	BRY233-19
B. amazonicus	86726	22343	Leticia/AM	Quebrada Yahuarcaca	Colombia	BRY240-19
B. cf. falcatus	19106	3027	Caicara del Orinoco/Bolivar	Rio Orinoco	Venezuela	BRY252-19
B. cf. falcatus	19108	3027	Caicara del Orinoco/Bolivar	Rio Orinoco	Venezuela	BRY251-19

Table S1. *Cont.*

Taxon	Voucher	Collection Code	City/State	Locality	Country	Accession Bold
B. cf. falcatus	19109	3027	Caicara del Orinoco/Bolivar	Rio Orinoco	Venezuela	BRY253-19
B. cf. falcatus	91257	25006	Novo Progresso/PA	Rio Jamanxin	Brazil	BRY250-19
B. chagrensis	18512	2750	Santa Rita Arriba/Colón	Río Llano Sucio	Panamá	BRY347-19
B. dentex	76111	19333	Babahoyo/Los Ríos	Rio Babahoyo	Equador	BRY254-19
B. dentex	76112	19333	Babahoyo/Los Ríos	Rio Babahoyo	Equador	BRY255-19
B. falcatus	7416	CPUFMT4495	Juruena/MT	Rio Juruena	Brazil	BRY001-16
B. falcatus	7417	CPUFMT4495	Juruena/MT	Rio Juruena	Brazil	BRY002-16
B. falcatus	7418	CPUFMT4495	Juruena/MT	Rio Juruena	Brazil	BRY003-16
B. falcatus	7419	CPUFMT4495	Juruena/MT	Rio Juruena	Brazil	BRY004-16
B. falcatus	7420	CPUFMT4495	Juruena/MT	Rio Juruena	Brazil	BRY005-16
B. falcatus	7421	CPUFMT4495	Juruena/MT	Rio Juruena	Brazil	BRY006-16
B. falcatus	7422	CPUFMT4495	Juruena/MT	Rio Juruena	Brazil	BRY007-16
B. falcatus	7423	CPUFMT4495	Juruena/MT	Rio Juruena	Brazil	BRY008-16
B. falcatus	7424	CPUFMT4495	Juruena/MT	Rio Juruena	Brazil	BRY009-16
B. falcatus	7425	Labgen	Juruena/MT	Rio Juruena	Brazil	BRY010-16
B. falcatus	7426	CPUFMT4495	Juruena/MT	Rio Juruena	Brazil	BRY011-16
B. falcatus	7427	CPUFMT4495	Juruena/MT	Rio Juruena	Brazil	BRY012-16
B. falcatus	7428	CPUFMT4495	Juruena/MT	Rio Juruena	Brazil	BRY013-16
B. falcatus	7429	CPUFMT4495	Juruena/MT	Rio Juruena	Brazil	BRY014-16
B. falcatus	7431	CPUFMT4495	Juruena/MT	Rio Juruena	Brazil	BRY016-16
B. falcatus	7432	CPUFMT4495	Juruena/MT	Rio Juruena	Brazil	BRY017-16
B. falcatus	7434	CPUFMT4495	Juruena/MT	Rio Juruena	Brazil	BRY019-16
B. falcatus	7435	CPUFMT4495	Juruena/MT	Rio Juruena	Brazil	BRY020-16
B. falcatus	7436	CPUFMT4495	Juruena/MT	Rio Juruena	Brazil	BRY021-16
B. falcatus	7869	Labgen	Juara/MT	Rio Arinos	Brazil	BRY023-16

Table S1. *Cont.*

Taxon	Voucher	Collection Code	City/State	Locality	Country	Accession Bold
B. falcatus	7873	Labgen	Juara/MT	Rio Arinos	Brazil	BRY026-16
B. falcatus	7875	Labgen	Juara/MT	Rio Arinos	Brazil	BRY028-16
B. falcatus	7877	Labgen	Juara/MT	Rio Arinos	Brazil	BRY029-16
B. falcatus	7878	Labgen	Juara/MT	Rio Arinos	Brazil	BRY030-16
B. falcatus	7879	Labgen	Juara/MT	Rio Arinos	Brazil	BRY031-16
B. falcatus	7880	Labgen	Juara/MT	Rio Arinos	Brazil	BRY032-16
B. falcatus	7882	Labgen	Juara/MT	Rio Arinos	Brazil	BRY034-16
B. falcatus	7884	Labgen	Juara/MT	Rio Arinos	Brazil	BRY036-16
B. falcatus	7885	Labgen	Juara/MT	Rio Arinos	Brazil	BRY037-16
B. falcatus	7892	Labgen	Juara/MT	Rio Arinos	Brazil	BRY043-16
B. falcatus	7893	Labgen	Juara/MT	Rio Arinos	Brazil	BRY044-16
B. falcatus	7894	Labgen	Juara/MT	Rio Arinos	Brazil	BRY045-16
B. falcatus	7895	Labgen	Juara/MT	Rio Arinos	Brazil	BRY046-16
B. falcatus	7896	Labgen	Juara/MT	Rio Arinos	Brazil	BRY047-16
B. falcatus	7898	Labgen	Juara/MT	Rio Arinos	Brazil	BRY048-16
B. falcatus	7901	Labgen	Juara/MT	Rio Arinos	Brazil	BRY051-16
B. falcatus	7909	Labgen	Juara/MT	Rio Arinos	Brazil	BRY058-16
B. falcatus	7953	Labgen	Juara/MT	Rio Arinos	Brazil	BRY062-16
B. falcatus	7954	Labgen	Juara/MT	Rio Arinos	Brazil	BRY063-16
B. falcatus	7956	Labgen	Juara/MT	Rio Arinos	Brazil	BRY064-16
B. falcatus	8538	CPUFMT4485	Barra do Garças/MT	Rio Araguaia	Brazil	BRY065-16
B. falcatus	8539	CPUFMT4485	Barra do Garças/MT	Rio Araguaia	Brazil	BRY066-16
B. falcatus	8540	Labgen	Paranatinga/MT	Rio culuene	Brazil	BRY067-16
B. falcatus	8541	Labgen	Paranatinga/MT	Rio culuene	Brazil	BRY068-16
B. falcatus	8543	Labgen	Paranatinga/MT	Rio culuene	Brazil	BRY070-16

Table S1. *Cont.*

Taxon	Voucher	Collection Code	City/State	Locality	Country	Accession Bold
B. falcatus	8544	Labgen	Paranatinga/MT	Rio culuene	Brazil	BRY071-16
B. falcatus	8545	Labgen	Paranatinga/MT	Rio culuene	Brazil	BRY072-16
B. falcatus	8546	Labgen	Paranatinga/MT	Rio culuene	Brazil	BRY073-16
B. falcatus	8547	Labgen	Paranatinga/MT	Rio culuene	Brazil	BRY074-16
B. falcatus	8548	Labgen	Paranatinga/MT	Rio culuene	Brazil	BRY075-16
B. falcatus	8549	Labgen	Paranatinga/MT	Rio culuene	Brazil	BRY076-16
B. falcatus	8550	Labgen	Paranatinga/MT	Rio culuene	Brazil	BRY077-16
B. falcatus	8551	Labgen	Paranatinga/MT	Rio culuene	Brazil	BRY078-16
B. falcatus	8552	Labgen	Paranatinga/MT	Rio culuene	Brazil	BRY079-16
B. falcatus	8555	Labgen	Barra do Garças/MT	Rio Araguaia	Brazil	BRY082-16
B. falcatus	8562	CPUFMT4498	Barra do Garças/MT	Rio Araguaia	Brazil	BRY089-16
B. falcatus	8618	CPUFMT4483	Campo Novo do Parecis/MT	Rio do sangue	Brazil	BRY106-16
B. falcatus	8619	CPUFMT4483	Campo Novo do Parecis/MT	Rio do sangue	Brazil	BRY107-16
B. falcatus	8620	CPUFMT4483	Campo Novo do Parecis/MT	Rio do sangue	Brazil	BRY108-16
B. falcatus	8621	CPUFMT4483	Campo Novo do Parecis/MT	Rio do sangue	Brazil	BRY109-16
B. falcatus	8695	Labgen	Sinop/MT	Rio Teles Pires	Brazil	BRY110-16
B. falcatus	8696	Labgen	Sinop/MT	Rio Teles Pires	Brazil	BRY111-16
B. falcatus	8697	Labgen	Sinop/MT	Rio Teles Pires	Brazil	BRY112-16
B. falcatus	8698	Labgen	Sinop/MT	Rio Teles Pires	Brazil	BRY113-16
B. falcatus	8699	Labgen	Sinop/MT	Rio Verde	Brazil	BRY114-16
B. falcatus	8700	Labgen	Sinop/MT	Rio Verde	Brazil	BRY115-16
B. falcatus	8701	Labgen	Sinop/MT	Rio Verde	Brazil	BRY116-16
B. falcatus	8702	Labgen	Vera/MT	Rio Celeste	Brazil	BRY117-16
B. falcatus	8703	Labgen	Vera/MT	Rio Celeste	Brazil	BRY118-16
B. falcatus	8704	Labgen	Vera/MT	Rio Celeste	Brazil	BRY119-16

Table S1. *Cont.*

Taxon	Voucher	Collection Code	City/State	Locality	Country	Accession Bold
B. falcatus	8705	Labgen	Vera/MT	Rio Celeste	Brazil	BRY120-16
B. falcatus	8706	Labgen	Vera/MT	Rio Celeste	Brazil	BRY121-16
B. falcatus	8707	Labgen	Vera/MT	Rio Celeste	Brazil	BRY122-16
B. falcatus	8708	Labgen	Sinop/MT	Rio Verde	Brazil	BRY123-16
B. falcatus	8709	Labgen	Sinop/MT	Rio Verde	Brazil	BRY124-16
B. falcatus	8710	Labgen	Sinop/MT	Rio Verde	Brazil	BRY125-16
B. falcatus	8711	Labgen	Sinop/MT	Rio Verde	Brazil	BRY126-16
B. falcatus	8760	CPUFMT4487	Paranaíta/MT	Rio Teles Pires	Brazil	BRY128-16
B. falcatus	8761	CPUFMT4487	Paranaíta/MT	Rio Teles Pires	Brazil	BRY129-16
B. falcatus	8762	CPUFMT4487	Paranaíta/MT	Rio Teles Pires	Brazil	BRY130-16
B. falcatus	8766	CPUFMT4487	Paranaíta/MT	Rio Teles Pires	Brazil	BRY134-16
B. falcatus	8769	CPUFMT4487	Paranaíta/MT	Rio Teles Pires	Brazil	BRY137-16
B. falcatus	8770	CPUFMT4487	Paranaíta/MT	Rio Teles Pires	Brazil	BRY138-16
B. falcatus	8840	Labgen	Rondônia/RO	Rio Madeira	Brazil	BRY147-16
B. falcatus	8841	Labgen	Rondônia/RO	Rio Madeira	Brazil	BRY148-16
B. falcatus	8870	CPUFMT4491	Luciara/MT	Rio Xavantinho	Brazil	BRY158-16
B. falcatus	8872	CPUFMT4491	Luciara/MT	Rio Xavantinho	Brazil	BRY159-16
B. falcatus	8873	CPUFMT4491	Luciara/MT	Rio Xavantinho	Brazil	BRY160-16
B. falcatus	8877	Labgen	Luciara/MT	Rio Xavantinho	Brazil	BRY162-16
B. falcatus	8878	Labgen	Luciara/MT	Rio Xavantinho	Brazil	BRY163-16
B. falcatus	8879	Labgen	Luciara/MT	Rio Xavantinho	Brazil	BRY164-16
B. falcatus	8883	CPUFMT4491	Luciara/MT	Rio Xavantinho	Brazil	BRY173-17
B. falcatus	8885	CPUFMT4491	Luciara/MT	Rio Xavantinho	Brazil	BRY175-17
B. falcatus	8892	CPUFMT4490	Luciara/MT	Rio Xavantinho	Brazil	BRY179-17
B. falcatus	8895	CPUFMT4484	Barra do Garças/MT	Rio Araguaia	Brazil	BRY166-16

Table S1. *Cont.*

Taxon	Voucher	Collection Code	City/State	Locality	Country	Accession Bold
B. falcatus	8896	CPUFMT4484	Barra do Garças/MT	Rio Araguaia	Brazil	BRY167-16
B. falcatus	8897	CPUFMT4484	Barra do Garças/MT	Rio Araguaia	Brazil	BRY168-16
B. falcatus	8898	CPUFMT4484	Barra do Garças/MT	Rio Araguaia	Brazil	BRY169-16
B. falcatus	8899	CPUFMT4484	Barra do Garças/MT	Rio Araguaia	Brazil	BRY170-16
B. falcatus	8900	CPUFMT4484	Barra do Garças/MT	Rio Araguaia	Brazil	BRY171-16
B. falcatus	8902	CPUFMT4484	Barra do Garças/MT	Rio Araguaia	Brazil	BRY180-17
B. falcatus	8903	CPUFMT4484	Barra do Garças/MT	Rio Araguaia	Brazil	BRY181-17
B. falcatus	8904	CPUFMT4484	Barra do Garças/MT	Rio Araguaia	Brazil	BRY182-17
B. falcatus	8905	CPUFMT4484	Barra do Garças/MT	Rio Araguaia	Brazil	BRY183-17
B. falcatus	8906	CPUFMT4484	Barra do Garças/MT	Rio Araguaia	Brazil	BRY184-17
B. falcatus	8908	CPUFMT4484	Barra do Garças/MT	Rio Araguaia	Brazil	BRY185-17
B. falcatus	8951	CPUFMT4486	Paranaíta/MT	Rio Teles Pires	Brazil	BRY186-17
B. falcatus	8952	CPUFMT4486	Paranaíta/MT	Rio Teles Pires	Brazil	BRY187-17
B. falcatus	8953	CPUFMT4486	Paranaíta/MT	Rio Teles Pires	Brazil	BRY188-17
B. falcatus	8954	CPUFMT4486	Paranaíta/MT	Rio Teles Pires	Brazil	BRY189-17
B. falcatus	8955	CPUFMT4486	Paranaíta/MT	Rio Teles Pires	Brazil	BRY190-17
B. falcatus	8957	CPUFMT4486	Paranaíta/MT	Rio Teles Pires	Brazil	BRY191-17
B. falcatus	8958	CPUFMT4486	Paranaíta/MT	Rio Teles Pires	Brazil	BRY192-17
B. falcatus	8967	CPUFMT4486	Paranaíta/MT	Rio Teles Pires	Brazil	BRY199-17
B. falcatus	9687	Labgen	São José do Rio Claro/MT	Rio Claro	Brazil	BRY207-19
B. falcatus	10212	Labgen	Cotriguaçu/MT	Rio Juruena	Brazil	BRY212-19
B. falcatus	10213	Labgen	Cotriguaçu/MT	Rio Juruena	Brazil	BRY213-19
B. falcatus	10214	Labgen	Cotriguaçu/MT	Rio Juruena	Brazil	BRY214-19
B. falcatus	10215	Labgen	Cotriguaçu/MT	Rio Juruena	Brazil	BRY215-19
B. falcatus	10216	Labgen	Cotriguaçu/MT	Rio Juruena	Brazil	BRY216-19

Table S1. *Cont.*

Taxon	Voucher	Collection Code	City/State	Locality	Country	Accession Bold
B. falcatus	10374	Labgen	São José do Rio Claro/MT	Rio Claro	Brazil	BRY364-19
B. falcatus	23017	4005	São Félix do Araguaia/MT	Lago Morto	Brazil	BRY257-19
B. falcatus	23019	4005	São Félix do Araguaia/MT	Lago Morto	Brazil	BRY258-19
B. falcatus	23036	4005	São Félix do Araguaia/MT	Lago Morto	Brazil	BRY259-19
B. falcatus	23037	4005	São Félix do Araguaia/MT	Lago Morto	Brazil	BRY256-19
B. falcatus	26273	5146	Ji-Paraná/RO	Rio Machado	Brazil	BRY260-19
B. falcatus	26281	5146	Ji-Paraná/RO	Rio Machado	Brazil	BRY261-19
B. falcatus	41280	5146	Ji-Paraná/RO	Rio Machado	Brazil	BRY262-19
B. falcatus	57395	13884	Itaituba/PA	Igarapé Urua	Brazil	BRY263-19
B. falcatus	66870	16141	Itaituba/PA	Igarapé Nambuaí	Brazil	BRY243-19
B. falcatus	66871	16141	Itaituba/PA	Igarapé Nambuaí	Brazil	BRY244-19
B. falcatus	67191	16242	Itaituba/PA	Igarapé São José	Brazil	BRY245-19
B. falcatus	67383	16421	Itaituba/PA	Igarapé da aldeia	Brazil	BRY246-19
B. falcatus	67547	16498	Itaituba/PA	Rio Tapajós	Brazil	BRY238-19
B. falcatus	67610	16601	Altamira/PA	Igarapé do Joé	Brazil	BRY239-19
B. falcatus	67730	16652	Altamira/PA	Rio Amazonas	Brazil	BRY319-19
B. ferox	37529	8100	Carlos Chagas/MG	Rio Mucuri	Brazil	BRY265-19
B. ferox	37534	8099	Carlos Chagas/MG	Rio Mucuri	Brazil	BRY264-19
B. ferox	47663	10181	Carlos Chagas/MG	Rio Mucuri	Brazil	BRY356-19
B. ferox	47664	10181	Carlos Chagas/MG	Rio Mucuri	Brazil	BRY357-19
B. gouldingi	8553	Labgen	Barra do Garças/MT	Rio Araguaia	Brazil	BRY080-16
B. gouldingi	8554	Labgen	Barra do Garças/MT	Rio Araguaia	Brazil	BRY081-16
B. gouldingi	8556	Labgen	Barra do Garças/MT	Rio Araguaia	Brazil	BRY083-16
B. gouldingi	8557	Labgen	Barra do Garças/MT	Rio Araguaia	Brazil	BRY084-16
B. gouldingi	8558	Labgen	Barra do Garças/MT	Rio Araguaia	Brazil	BRY085-16

Table S1. *Cont.*

Taxon	Voucher	Collection Code	City/State	Locality	Country	Accession Bold
B. gouldingi	8559	Labgen	Barra do Garças/MT	Rio Araguaia	Brazil	BRY086-16
B. gouldingi	8560	Labgen	Barra do Garças/MT	Rio Araguaia	Brazil	BRY087-16
B. gouldingi	8561	Labgen	Barra do Garças/MT	Rio Araguaia	Brazil	BRY088-16
B. gouldingi	8563	Labgen	Barra do Garças/MT	Rio Araguaia	Brazil	BRY090-16
B. gouldingi	8564	Labgen	Barra do Garças/MT	Rio Araguaia	Brazil	BRY091-16
B. gouldingi	8565	Labgen	Barra do Garças/MT	Rio Araguaia	Brazil	BRY092-16
B. gouldingi	8567	Labgen	Barra do Garças/MT	Rio Araguaia	Brazil	BRY094-16
B. gouldingi	8568	Labgen	Barra do Garças/MT	Rio Araguaia	Brazil	BRY095-16
B. gouldingi	8569	Labgen	Barra do Garças/MT	Rio Araguaia	Brazil	BRY096-16
B. gouldingi	8570	Labgen	Barra do Garças/MT	Rio Araguaia	Brazil	BRY097-16
B. gouldingi	8571	Labgen	Barra do Garças/MT	Rio Araguaia	Brazil	BRY098-16
B. gouldingi	8572	Labgen	Barra do Garças/MT	Rio Araguaia	Brazil	BRY099-16
B. gouldingi	8573	Labgen	Barra do Garças/MT	Lago quatro bocas-Araguaina	Brazil	BRY100-16
B. gouldingi	8574	Labgen	Barra do Garças/MT	Rio Araguaia	Brazil	BRY101-16
B. gouldingi	8575	Labgen	Barra do Garças/MT	Rio Araguaia	Brazil	BRY102-16
B. gouldingi	8576	Labgen	Barra do Garças/MT	Rio Araguaia	Brazil	BRY103-16
B. gouldingi	8577	Labgen	Barra do Garças/MT	Rio Araguaia	Brazil	BRY104-16
B. gouldingi	8578	Labgen	Barra do Garças/MT	Rio Araguaia	Brazil	BRY105-16
B. gouldingi	8874	CPUFMT4490	Luciara/MT	Rio Xavantinho	Brazil	BRY161-16
B. gouldingi	8882	Labgen	Luciara/MT	Rio Xavantinho	Brazil	BRY172-17
B. gouldingi	8884	CPUFMT4490	Luciara/MT	Rio Xavantinho	Brazil	BRY174-17
B. gouldingi	19203	3130	Cocalinho/MT	Lagoa da Égua	Brazil	BRY266-19
B. henni	91123	24255	Piedra Moler/Valle del Cauca	Rio La Veja	Colombia	BRY270-19
B. henni	91124	24255	Piedra Moler/Valle del Cauca	Rio La Veja	Colombia	BRY271-19
B. henni	91189	24248	Zarzal/Valle del Cauca	Rio La Paila	Colombia	BRY267-19

Table S1. *Cont.*

Taxon	Voucher	Collection Code	City/State	Locality	Country	Accession Bold
B. henni	91190	24248	Zarzal/Valle del Cauca	Rio La Paila	Colombia	BRY268-19
B. henni	91191	24248	Zarzal/Valle del Cauca	Rio La Paila	Colombia	BRY269-19
B. hilarii	9579	Labgen	Cáceres/MT	Rio Paraguai	Brazil	BRY203-19
B. hilarii	9580	Labgen	Cáceres/MT	Rio Paraguai	Brazil	BRY204-19
B. hilarii	9581	Labgen	Cáceres/MT	Rio Paraguai	Brazil	BRY205-19
B. hilarii	9582	Labgen	Cáceres/MT	Rio Paraguai	Brazil	BRY206-19
B. hilarii	10410	Labgen	Unknown	Rio Jauru	Brazil	BRY365-19
B. hilarii	24811	4676	Santo Antônio do Leverger/MT	Rio Cuiabá	Brazil	BRY272-19
B. hilarii	24812	Labgen	Santo Antônio do Leverger/MT	Rio Paraguai	Brazil	BRY273-19
B. hilarii	24813	4676	Santo Antônio do Leverger/MT	Rio Cuiabá	Brazil	BRY274-19
B. insignis	16080	2369	Campos dos Goytacazes/RJ	Rio Paraíba do Sul	Brazil	BRY275-19
B. insignis	18976	2854	Unknown	Unknown	Brazil	BRY276-19
B. melanopterus	38907	9778	Iquitos	Rio Amazonas	Peru	BRY278-19
B. melanopterus	38908	9778	Iquitos	Rio Amazonas	Peru	BRY279-19
B. melanopterus	38909	9778	Iquitos	Rio Amazonas	Peru	BRY280-19
B. melanopterus	38910	9778	Iquitos	Rio Amazonas	Peru	BRY281-19
B. melanopterus	38911	9778	Iquitos	Rio Amazonas	Peru	BRY282-19
B. melanopterus	86829	22423	Leticia/AM	Quebrada La Ponderosa	Colombia	BRY283-19
B. melanopterus	86830	22423	Leticia/AM	Quebrada La Ponderosa	Colombia	BRY284-19
B. melanopterus	86831	22423	Leticia/AM	Quebrada La Ponderosa	Colombia	BRY285-19
B. melanopterus	86832	22423	Leticia/AM	Quebrada La Ponderosa	Colombia	BRY286-19
B. melanopterus	86833	22423	Leticia/AM	Quebrada La Ponderosa	Colombia	BRY287-19
B. melanopterus	87537	22516	Leticia/AM	Lago Yahuarcaca	Colombia	BRY241-19
B. melanopterus	87538	22516	Leticia/AM	Lago Yahuarcaca	Colombia	BRY242-19
B. melanopterus	88035	22711	Tabatinga/AM	Igarapé Xingu	Brazil	BRY247-19

Table S1. *Cont.*

Taxon	Voucher	Collection Code	City/State	Locality	Country	Accession Bold
B. moorei	70172	12818	Unknown	Rio Magdalena	Colombia	BRY288-19
B. moorei	70173	12818	Unknown	Rio Magdalena	Colombia	BRY289-19
B. moorei	70174	12818	Unknown	Rio Magdalena	Colombia	BRY290-19
B. nattereri	18967	2851	Unknown	Unknown	Brazil	BRY248-19
B. nattereri	18968	2851	Unknown	Unknown	Brazil	BRY249-19
B. nattereri	35837	7271	Bela Vista de Goias/GO	Rio Arapuca	Brazil	BRY294-19
B. nattereri	37541	8101	Carrancas/MG	Rio Capivari	Brazil	BRY291-19
B. nattereri	37544	8101	Carrancas/MG	Rio Capivari	Brazil	BRY292-19
B. nattereri	37545	8101	Carrancas/MG	Rio Capivari	Brazil	BRY293-19
B. opalinus	18989	2860	Unknown	Unknown	Colombia	BRY295-19
B. opalinus	18990	2861	Unknown	Unknown	Colombia	BRY296-19
B. opalinus	18991	2862	Unknown	Unknown	Colombia	BRY297-19
B. opalinus	29005	6303	Areias/SP	Rio Itagaçaba	Brazil	BRY299-19
B. opalinus	29006	6303	Areias/SP	Rio Itagaçaba	Brazil	BRY298-19
B. orbignyanus	10480	2691	Pirassununga/SP	Piscicultura CEPTA	Brazil	BRY300-19
B. orbignyanus	18004	2746	Pirassununga/SP	Piscicultura	Brazil	BRY301-19
B. orbignyanus	18005	2746	Pirassununga/SP	Piscicultura	Brazil	BRY302-19
B. orbignyanus	18006	2746	Pirassununga/SP	Piscicultura	Brazil	BRY303-19
B. orbignyanus	18007	2746	Pirassununga/SP	Piscicultura	Brazil	BRY304-19
B. orthotaenia	4215	249	Três Marias - MG	Rio São Francisco	Brazil	BRY305-19
B. orthotaenia	18964	2850	Unknown	Unknown	Brazil	BRY277-19
B. orthotaenia	28361	5844	Três Marias - MG	Piscicultura da CODEVASF	Brazil	BRY306-19
B. pesu	7430	CPUFMT4492	Juruena/MT	Rio Juruena	Brazil	BRY015-16
B. pesu	7433	CPUFMT4492	Juruena/MT	Rio Juruena	Brazil	BRY018-16
B. pesu	7874	Labgen	Juara/MT	Rio Arinos	Brazil	BRY027-16

Table S1. *Cont.*

Taxon	Voucher	Collection Code	City/State	Locality	Country	Accession Bold
B. pesu	7883	Labgen	Juara/MT	Rio Arinos	Brazil	BRY035-16
B. pesu	7888	Labgen	Juara/MT	Rio Arinos	Brazil	BRY040-16
B. pesu	7899	Labgen	Juara/MT	Rio Arinos	Brazil	BRY049-16
B. pesu	7900	Labgen	Juara/MT	Rio Arinos	Brazil	BRY050-16
B. pesu	7902	Labgen	Juara/MT	Rio Arinos	Brazil	BRY052-16
B. pesu	7903	Labgen	Juara/MT	Rio Arinos	Brazil	BRY053-16
B. pesu	7904	Labgen	Juara/MT	Rio Arinos	Brazil	BRY054-16
B. pesu	7905	Labgen	Juara/MT	Rio Arinos	Brazil	BRY055-16
B. pesu	7910	Labgen	Juara/MT	Rio Arinos	Brazil	BRY059-16
B. pesu	7911	Labgen	Juara/MT	Rio Arinos	Brazil	BRY060-16
B. pesu	8758	CPUFMT4488	Paranaíta/MT	Rio Teles Pires	Brazil	BRY127-16
B. pesu	8763	CPUFMT4488	Paranaíta/MT	Rio Teles Pires	Brazil	BRY131-16
B. pesu	8764	Labgen	Paranaíta/MT	Rio Teles Pires	Brazil	BRY132-16
B. pesu	8765	CPUFMT4488	Paranaíta/MT	Rio Teles Pires	Brazil	BRY133-16
B. pesu	8767	CPUFMT4488	Paranaíta/MT	Rio Teles Pires	Brazil	BRY135-16
B. pesu	8768	CPUFMT4488	Paranaíta/MT	Rio Teles Pires	Brazil	BRY136-16
B. pesu	8771	CPUFMT4488	Paranaíta/MT	Rio Teles Pires	Brazil	BRY139-16
B. pesu	8772	CPUFMT4488	Paranaíta/MT	Rio Teles Pires	Brazil	BRY140-16
B. pesu	8773	CPUFMT4488	Paranaíta/MT	Rio Teles Pires	Brazil	BRY141-16
B. pesu	8774	CPUFMT4488	Paranaíta/MT	Rio Teles Pires	Brazil	BRY142-16
B. pesu	8775	CPUFMT4488	Paranaíta/MT	Rio Teles Pires	Brazil	BRY143-16
B. pesu	8776	CPUFMT4488	Paranaíta/MT	Rio Teles Pires	Brazil	BRY144-16
B. pesu	8777	CPUFMT4488	Paranaíta/MT	Rio Teles Pires	Brazil	BRY145-16
B. pesu	8778	CPUFMT4488	Paranaíta/MT	Rio Teles Pires	Brazil	BRY146-16
B. pesu	8842	Labgen	Rondônia/RO	Rio Madeira	Brazil	BRY149-16

Table S1. *Cont.*

Taxon	Voucher	Collection Code	City/State	Locality	Country	Accession Bold
B. pesu	8843	Labgen	Rondônia/RO	Rio Madeira	Brazil	BRY150-16
B. pesu	8844	Labgen	Rondônia/RO	Rio Madeira	Brazil	BRY151-16
B. pesu	8846	Labgen	Rondônia/RO	Rio Madeira	Brazil	BRY153-16
B. pesu	8847	Labgen	Rondônia/RO	Rio Madeira	Brazil	BRY154-16
B. pesu	8848	Labgen	Rondônia/RO	Rio Madeira	Brazil	BRY155-16
B. pesu	8849	Labgen	Rondônia/RO	Rio Madeira	Brazil	BRY156-16
B. pesu	8850	Labgen	Rondônia/RO	Rio Madeira	Brazil	BRY157-16
B. pesu	8889	CPUFMT4493	Luciara/MT	Rio Xavantinho	Brazil	BRY178-17
B. pesu	8894	CPUFMT4493	Luciara/MT	Rio Xavantinho	Brazil	BRY165-16
B. pesu	8959	CPUFMT4494	Paranaíta/MT	Rio Teles Pires	Brazil	BRY193-17
B. pesu	8960	CPUFMT4494	Paranaíta/MT	Rio Teles Pires	Brazil	BRY194-17
B. pesu	8961	CPUFMT4494	Paranaíta/MT	Rio Teles Pires	Brazil	BRY195-17
B. pesu	8962	CPUFMT4494	Paranaíta/MT	Rio Teles Pires	Brazil	BRY196-17
B. pesu	8965	CPUFMT4494	Paranaíta/MT	Rio Teles Pires	Brazil	BRY198-17
B. pesu	8966	CPUFMT4494	Paranaíta/MT	Rio Teles Pires	Brazil	BRY367-19
B. pesu	8970	CPUFMT4494	Paranaíta/MT	Rio Teles Pires	Brazil	BRY201-17
B. pesu	10208	Labgen	Cotriguaçu/MT	Rio Juruena	Brazil	BRY208-19
B. pesu	10209	Labgen	Cotriguaçu/MT	Rio Juruena	Brazil	BRY209-19
B. pesu	10210	Labgen	Cotriguaçu/MT	Rio Juruena	Brazil	BRY210-19
B. pesu	10211	Labgen	Cotriguaçu/MT	Rio Juruena	Brazil	BRY211-19
B. pesu	12848	1724	Manaus/AM	Rio Tarumã	Brazil	BRY307-19
B. pesu	15715	2250	Caicara del Orinoco /Bolivar	Rio Orinoco	Venezuela	BRY308-19
B. pesu	15806	2286	Caicara del Orinoco /Bolivar	Rio Orinoco	Venezuela	BRY309-19
B. pesu	15807	2286	Caicara del Orinoco /Bolivar	Rio Orinoco	Venezuela	BRY310-19
B. pesu	24236	4406	Barcelos/AM	Rio Negro	Brazil	BRY335-19

Table S1. *Cont.*

Taxon	Voucher	Collection Code	City/State	Locality	Country	Accession Bold
B. pesu	24237	4406	Barcelos/AM	Rio Negro	Brazil	BRY336-19
B. pesu	24238	4406	Barcelos/AM	Rio Negro	Brazil	BRY337-19
B. pesu	24239	4406	Barcelos/AM	Rio Negro	Brazil	BRY338-19
B. pesu	24240	4406	Barcelos/AM	Rio Negro	Brazil	BRY339-19
B. pesu	26778	5290	Laranjal do Jari/AP	Igarapé Uiratapuru	Brazil	BRY311-19
B. pesu	26779	5290	Laranjal do Jari/AP	Igarapé Uiratapuru	Brazil	BRY312-19
B. pesu	26780	5290	Laranjal do Jari/AP	Igarapé Uiratapuru	Brazil	BRY313-19
B. pesu	26930	5320	Laranjal do Jari/AP	Rio Jari	Brazil	BRY314-19
B. pesu	26931	5320	Laranjal do Jari/AP	Rio Jari	Brazil	BRY315-19
B. pesu	32390	6877	São Gabriel da Cachoeira/AM	Rio Negro	Brazil	BRY316-19
B. pesu	32391	6877	São Gabriel da Cachoeira/AM	Rio Negro	Brazil	BRY317-19
B. pesu	47942	12713	Cocalinho/MT	Rio Araguaia	Brazil	BRY318-19
B. pesu	57120	13781	Itaituba/PA	Rio Tapajós	Brazil	BRY328-19
B. pesu	57219	13815	Itaituba/PA	Rio Tapajós	Brazil	BRY329-19
B. pesu	63791	15480	Caracaraí/RR	Cachoeira do Bem Querer	Brazil	BRY330-19
B. pesu	65639	15934	Canarana/MT	Rio Culuene	Brazil	BRY331-19
B. pesu	65640	15934	Canarana/MT	Rio Culuene	Brazil	BRY332-19
B. pesu	66963	16204	Itaituba/PA	Rio Tracuá	Brazil	BRY333-19
B. pesu	67390	16426	Itaituba/PA	Igarapé da aldeia	Brazil	BRY334-19
B. pesu	67890	16725	Altamira/PA	Rio Xingu	Brazil	BRY320-19
B. pesu	67891	16725	Altamira/PA	Rio Xingu	Brazil	BRY021-16
B. pesu	67892	16725	Altamira/PA	Rio Xingu	Brazil	BRY322-19
B. pesu	67893	16725	Altamira/PA	Rio Xingu	Brazil	BRY323-19
B. pesu	70459	17683	São Félix do Xingu/PA	Igarapé em Ilha no rio Xingu	Brazil	BRY340-19
B. pesu	80764	20451	Almeirim/PA	Rio Jari	Brazil	BRY342-19

Table S1. *Cont.*

Taxon	Voucher	Collection Code	City/State	Locality	Country	Accession Bold
B. pesu	80765	20451	Almeirim/Pará	Rio Jari	Brazil	BRY343-19
B. pesu	80766	20451	Almeirim/Pará	Rio Jari	Brazil	BRY344-19
B. pesu	80767	20451	Almeirim/PA	Rio Jari	Brazil	BRY345-19
B. pesu	80782	20457	Laranjal do Jari/AP	Rio Iratapuru	Brazil	BRY341-19
B. pesu	81886	20957	Almeirim/PA	Rio Jari	Brazil	BRY324-19
B. pesu	81887	20957	Almeirim/PA	Rio Jari	Brazil	BRY325-19
B. pesu	81888	20957	Almeirim/PA	Rio Jari	Brazil	BRY326-19
B. pesu	81889	20957	Almeirim/PA	Rio Jari	Brazil	BRY327-19
B. petrosus	18501	2764	Unknown	Unknown	Panamá	BRY351-19
B. petrosus	18502	2764	Unknown	Unknown	Panamá	BRY352-19
B. petrosus	18504	2750	Santa Rita Arriba/Colón	Río Llano Sucio	Panamá	BRY348-19
B. petrosus	18505	2750	Santa Rita Arriba/Colón	Río Llano Sucio	Panamá	BRY349-19
B. petrosus	18506	2750	Santa Rita Arriba/Colón	Río Llano Sucio	Panamá	BRY350-19
B. petrosus	18511	2750	Santa Rita Arriba/Colón	Río Llano Sucio	Panamá	BRY346-19
B. sp. equador	86244	23632	Santa Marta/El Oro	Rio Santa Marta	Equador	BRY353-19
B. sp. equador	86245	23632	Santa Marta/El Oro	Rio Santa Marta	Equador	BRY354-19
B. sp. equador	86246	23632	Santa Marta/El Oro	Rio Santa Marta	Equador	BRY355-19
C. deuterodon	43883	9341	Pampa de Hospital/Tumbes	Quebrada Cabuyal	Peru	BRY362-19
C. deuterodon	43884	9341	Pampa de Hospital/Tumbes	Quebrada Cabuyal	Peru	BRY363-19
C. deuterodon	43961	9369	Tumbes	Rio Tumbes	Peru	BRY361-19
C. deuterodon	43962	9369	Tumbes	Rio Tumbes	Peru	BRY362-19
H. wheatlandi	75472	7621	Ferros/MG	Rio Santo Antônio	Brazil	BRY358-19
H. wheatlandi	75473	7621	Ferros/MG	Rio Santo Antônio	Brazil	BRY359-19
