# Supplementary figures and images for: DNA Barcoding Reveals High Levels of Divergence among Mitochondrial Lineages of Brycon (Characiformes, Bryconidae)
Source: Genes (Basel). 2019 Aug 23;10(9):639. doi: 10.3390/genes10090639 (PMC6769914; doi:10.3390/genes10090639)

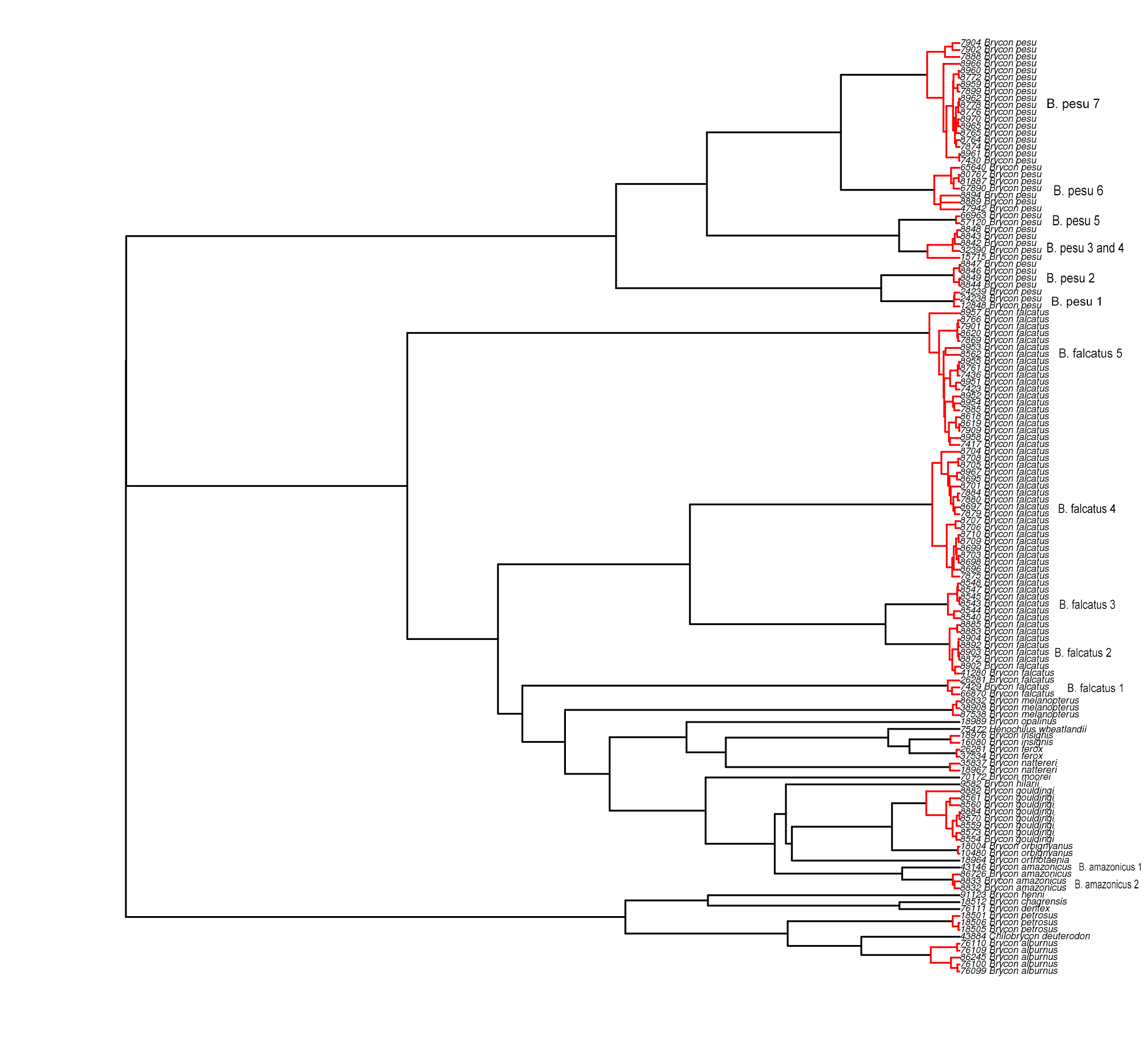

Supplement: Supplementary file 1 [file genes-10-00639-s001.zip › Fig S3 GMYC.tif]
